# Sigmund Freud (1856–1939)

**DOI:** 10.1007/s00415-020-09972-4

**Published:** 2020-06-08

**Authors:** Andrzej Grzybowski, Joanna Żołnierz

**Affiliations:** 1 Department of Ophthalmology, University of Warmia and Mazury, Olsztyn, Poland; 2 Institute for Research in Ophthalmology, Gorczyczewskiego 2/3, 61-553 Poznan, Poland; 3 Interfaculty Center for Didactics, Medical University of Lublin, Lublin, Poland

Although best known for his pioneering work in psychiatry and psychoanalysis, Sigmund Freud began his medical career in neurology.

**Fig.1:**
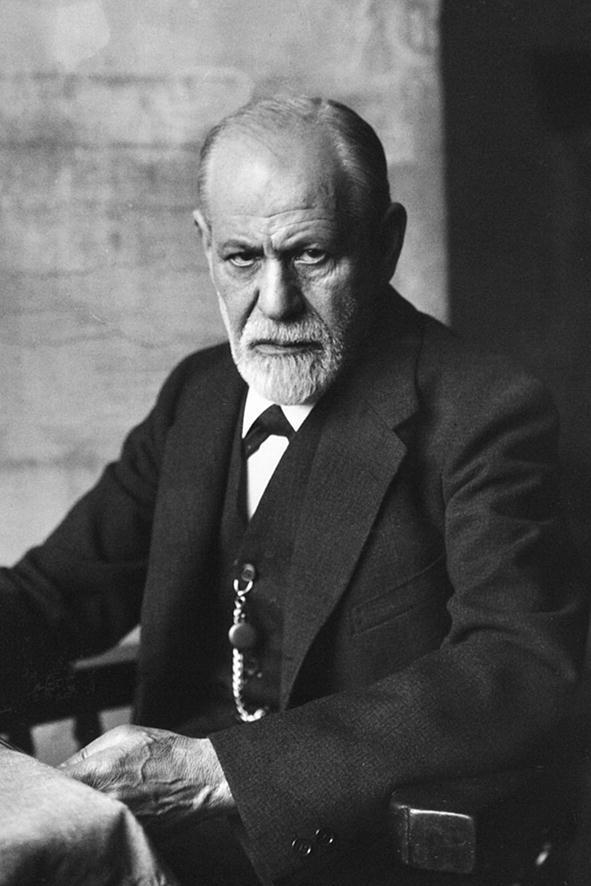
Sigmund Freud (1856–1939) Source: https://www.pxfuel.com/en/free-photo-joqrd

Sigismund Schlomo Freud (Fig. 1) was born on May 6, 1856, in Freiberg—a small town in Moravia (today called Příbor, Czech Republic). Freud came from a nonaffluent Jewish family of wool merchants. In 1859**–**1860, the Freud family first moved from Freiberg to Leipzig, and then to Vienna, where Freud spent most of his life [1, 2].

In 1873, Freud graduated with distinction from gymnasium and in the same year he began studying medicine at the University of Vienna. Three years later, he started working in the physiological laboratory under the direction of Ernst Wilhelm von Brücke. Freud was entrusted to research the histology of the nervous system, with which, to the delight of his mentor, he coped very well. As a result, Freud continued his research and worked intermittently in the laboratory from 1876 to 1882 [1, 2]. In the physiological laboratory, Freud examined the spinal cord of fish of the species *Ammocoetes petromyzon* and described the phylogenetic origin of posterior root ganglia based on an analysis of the fish’s spinal cord [3]. Moreover, he described the structure of the eel's lobe-shaped organ [4] and the origin of both nerve root and dorsal root ganglion [5].

In 1881, Freud graduated from the University of Vienna. Brücke, concerned about Freud's unfavorable financial situation, convinced him to resign from his research work in the laboratory and to engage in medical practice. In 1882, according to his mentor's recommendation, Freud started working at the General Hospital in Vienna as a clinical assistant. In this same year, he also got engaged to Martha Bernays [2]. Around that time, Freud additionally worked on a few clinical problems. He described the process of nervous system damage in a patient suffering from scurvy [6] and described a new technique of staining nerve fibers, which he had discovered [7]. Thanks to this method, Freud was able to examine and describe the structure of the medulla oblongata, the fibrillary tracts between the medulla oblongata and the cerebellum, and the origin of the nerve fibers [8].

In 1885, Freud was appointed Lecturer on Nervous Diseases at the University in Vienna. In this way, he obtained the opportunity to organize funding to visit Jean–Martin Charcot at the Salpêtrière Hospital. Freud went to Paris for the longed-for internship, where he spent several months. Charcot's claims were radically different from those accepted by the Viennese medical community, to which young Freud belonged. Particularly Charcot's statement on the psychological but not organic basis of hysteria had a huge impact on Freud's further research. Since his stay in Paris, Freud became increasingly interested in theories explaining the functioning of the human mind, which ultimately led to the foundation of a new psychological current—psychoanalysis [2]. After his stay in Paris, Freud spent a few weeks in Berlin, where he learned more about neurological diseases of childhood. This experience was necessary for him to lead the Department of Nervous Diseases of Children in Vienna. After returning to Vienna in 1886, Freud started working as a neurologist at the Kassowitz Institute and married Martha. He also tried to present the conclusions of his visit to Paris, but his views on the occurrence of hysteria in men and the appearance of hysterical paralysis by suggestion met strong opposition from the Viennese medical society. This resulted in their forbidding Freud to conduct research at the Institute of Cerebral Anatomy, and their reluctance to listen to Freud's lectures. This led him to withdraw from academic activity [2].

In 1886, Freud also resigned from work in the General Hospital in Vienna for private practice focused on neurosis treatment [1]. Although in the years 1886**–**1891 he devoted most of his time seeking gainful employment to maintain his growing family, he managed to publish significant works in the field of neurology [2]. One of them entitled: *On Aphasia: A Critical Study* (1891) was a result of already extensive clinical experience [9], included many clinical cases of aphasia and introduced the term “agnosia”. He also conducted work on children with cerebral palsy, which continued in future years [10]. Publications on aphasia and childhood cerebral palsy, the latest of Freud’s works in the field of neurology, are of great value even today.

In the following years of Freud's activities strictly concerned the theory of psychoanalysis and focused on cooperation with Josef Breuer, a Viennese family doctor and Freud's friend. In 1893, their *Preliminary Report* was published, and 2 years later, their *Studies on Hysteria* [2]. As a result of conflict, their friendship fell apart, and Freud undertook work on psychoanalysis and published many works, of which *The Interpretation of Dreams* (1900) and *A General Introduction to Psychoanalysis* (1917) are considered by some to be the most important [2].

In 1938, because of growing hostility toward Jews, Freud and his family fled to England, where he spent the rest of his life. Freud, struggling with oral cancer, died on September 23, 1939, 2 days after receiving morphine injections [1]. Freud's funeral took place at Golders Green Crematorium, and his ashes were laid at the East Columbarium in London.
